# Supplementary material for: Amazon rainforest adjusts to long-term experimental drought
Source: Nat Ecol Evol. 2025 May 15;9(6):970–9. doi: 10.1038/s41559-025-02702-x (PMC12148936; doi:10.1038/s41559-025-02702-x)
Supplement: Supplementary file 1 — Supplementary Figs. 1–9 and Tables 1 and 2. [file 41559_2025_2702_MOESM1_ESM.pdf]

nature ecology & evolution

Supplementary information https://doi.org/10.1038/s41559-025-02702-x

Amazon rainforest adjusts to long-term experimental drought

In the format provided by the authors and unedited

SUPPLEMENTARY INFORMATION

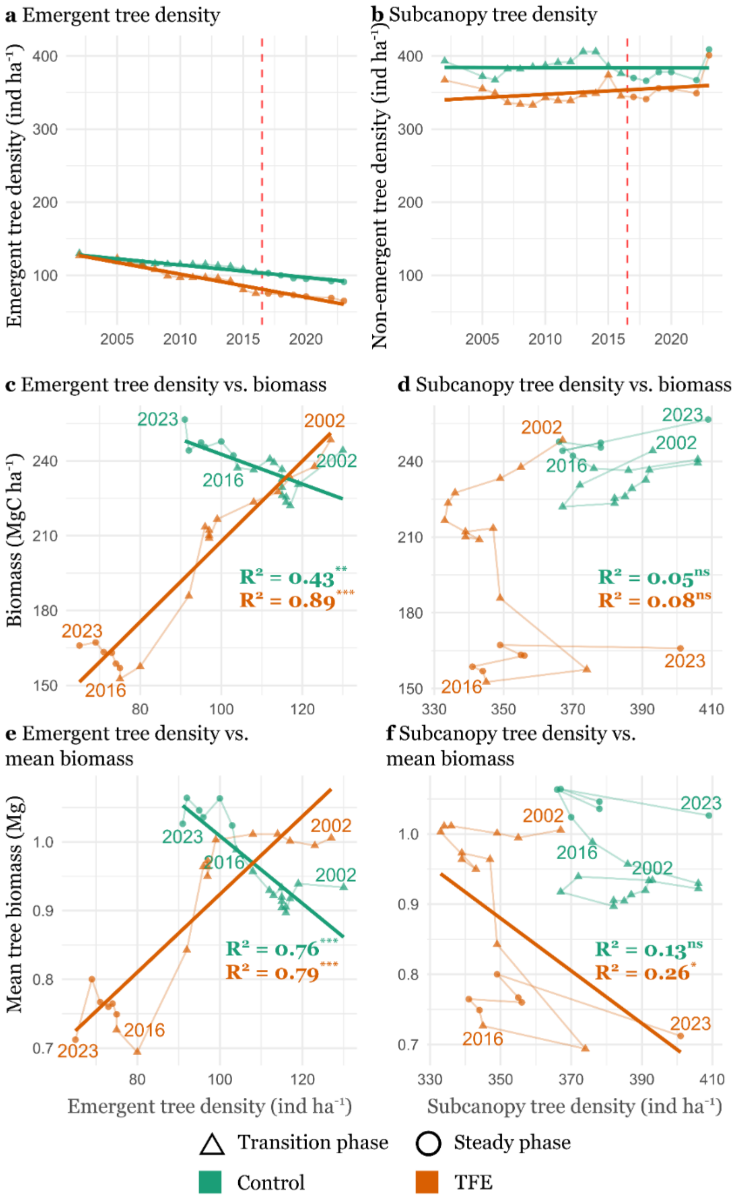

Figure S1. Emergent trees density drive patterns in forest biomass

Time series of emergent and subcanopy tree density (a and b, respectively) and their relationship with total above ground wood biomass (c, d) and mean above ground wood biomass (e, f). In a and b, red dotted line represents the approximate time at which the TFE plot changed from transition to steady phase. The two phases are represented by the shape of the points, triangles referring to transition phase and points to the steady phase. In c, d, e and f, consecutive years are connected by a line in the scatterplot, showing the first year of experiment (2002), the year after which the forest entered the steady phase (2016) and the latest year of experiment in our dataset (2023). Regression lines are represented when statistically significant and R^2 reported by linear models for each plot are also shown jointly with the model significance. Signif. codes: '***': P < 0.001; '**': P < 0.01; '*': P < 0.05 'ns': P > 0.05.

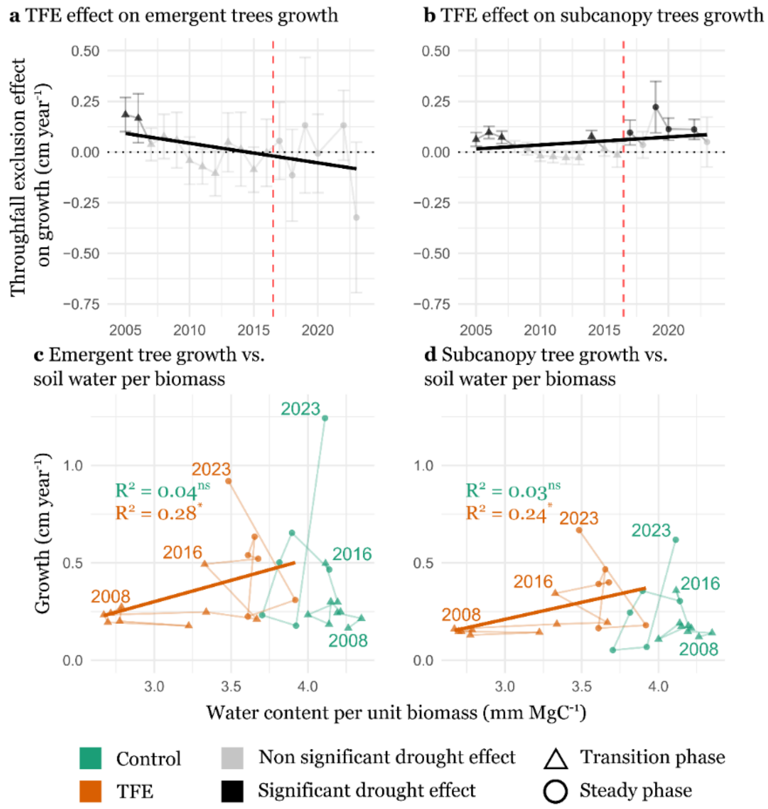

Figure S2. Subcanopy trees present higher growth after multi-decadal drought

Time series of plot effect on emergent and subcanopy annual stem growth reported by linear models (a and b) and the relationship between emergent and subcanopy annual stem growth with total above ground wood biomass (c and d). In a and b, red dotted line represents the approximate time at which the TFE plot changed from transition to steady phase. The two phases are represented by the shape of the points, triangles referring to transition phase (2002 to 2016) and points to the steady phase (2016-2023). In e and f, consecutive years are connected by a line in the scatterplot, showing the first year with soil water content per unit biomass and growth (2008), the year after which the forest entered the steady phase (2016) and the latter year of experiment (2023). Statistically significant linear relationships are represented and R^2 reported by linear models for each plot are also shown jointly with the model significance. Signif. codes: '***': $P < 0.001$; '**': $P < 0.01$; '*': $P < 0.05$ 'ns': $P > 0.05$.

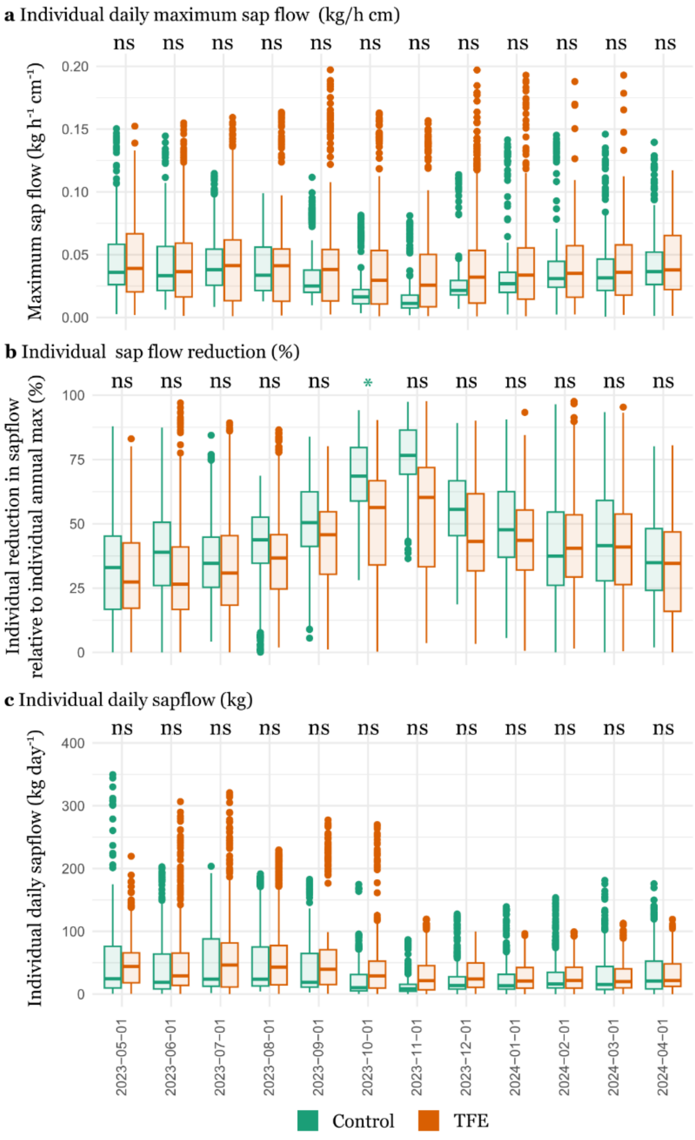

Figure S3. Tree transpiration month by month

Transpiration (measured as sap flow) differences between trees from the Through Fall Exclusion plot (TFE) and the Control plot. Daily maximum sap flow per unit sapwood represented for each plot and month (represented by the quantile 90%) (a); percentage of reduction of maximum daily sap flow compared to annual maxima (b) and total daily sap flow (c) from May 2023 to December 2023. Statistical significance was tested by means of linear mixed models (see Methods). Signif. codes: '***': P < 0.001; '**': P < 0.01; '*': P < 0.05 'ns': P > 0.05. Boxplots represent first, second and third quartiles and whiskers represent maxima and minima.

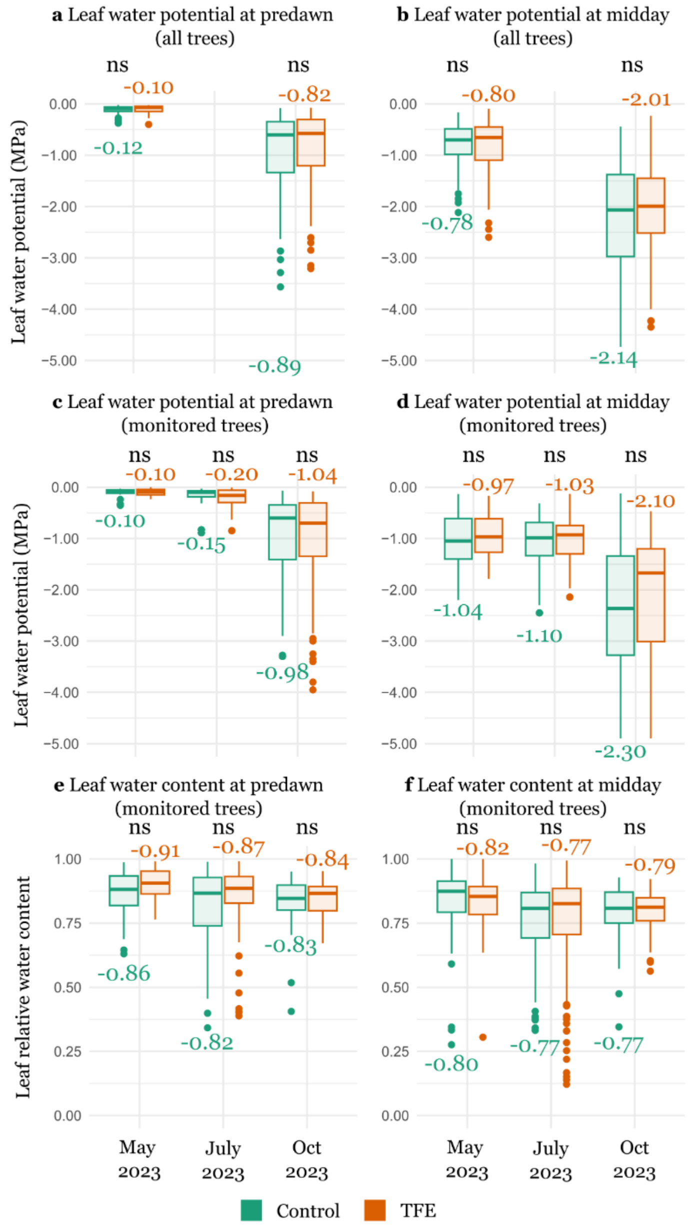

Figure S4. Tree leaf water status

Leaf water potential and water content measured in individual trees from the Through Fall Exclusion plot (TFE) and the Control plot. Water potential was measured for all trees (>352 trees) at the peak of the wet (05/2023) and the dry (10/2023) season. For a subset of this large sample of trees (i.e., the 42 monitored trees, see table S1), water contents were also measured and an extra campaign at the beginning of the dry season (07/2023) was added. Predawn samples were taken from 4 am to 6 am and show water status at equilibrium with soil water. Midday samples were taken from 11:45 am to 2 pm and show water status at maximum stress (maximum atmospheric water demand). Mean values for each plot and campaign are shown. Statistical significance was tested by means of linear mixed models (see Methods). Signif. codes: '***': $P < 0.001$; '**': $P < 0.01$; '*': $P < 0.05$ 'ns': $P > 0.05$. Boxplots represent first, second and third quartiles and whiskers represent maxima and minima.

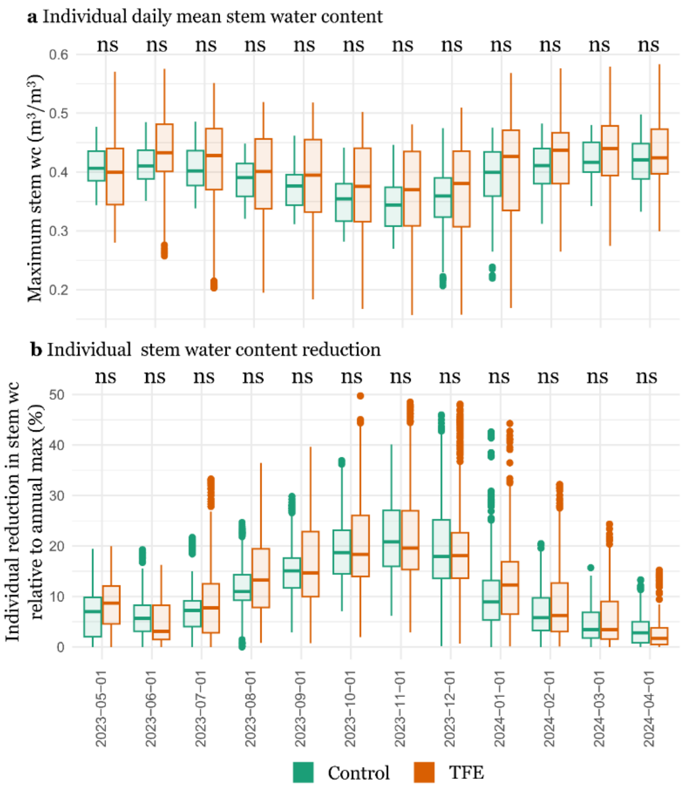

Figure S5. Monthly difference in stem water content

Stem water content differences between trees from the Through Fall Exclusion plot (TFE) and the Control plot (21 trees per plot). Daily maximum sap flow per unit sapwood represented for each plot and month (represented by the quantile 90%) (a); reduction in maximum daily sap flow from annual maxima (b) from May 2023 to December 2023. Statistical significance was tested by means of linear mixed models (see Methods). Signif. codes: '***': P < 0.001; '**': P < 0.01; '*': P < 0.05 'ns': P > 0.05. Boxplots represent first, second and third quartiles and whiskers represent maxima and minima.

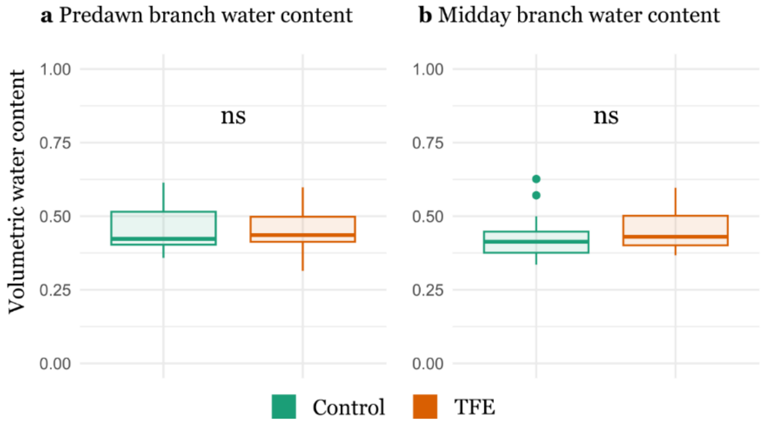

Figure S6. Branch volumetric water content during the peak of the dry season

Branch volumetric water content sampled in the dry season (October 2023) in individuals from the Through Fall Exclusion plot (TFE) and from the Control plot (21 trees per plot). Statistical significance was tested by means of linear mixed models (see Methods). Signif. codes: '***': P < 0.001; '**': P < 0.01; '*': P < 0.05 'ns': P > 0.05. Boxplots represent first, second and third quartiles and whiskers represent maxima and minima.

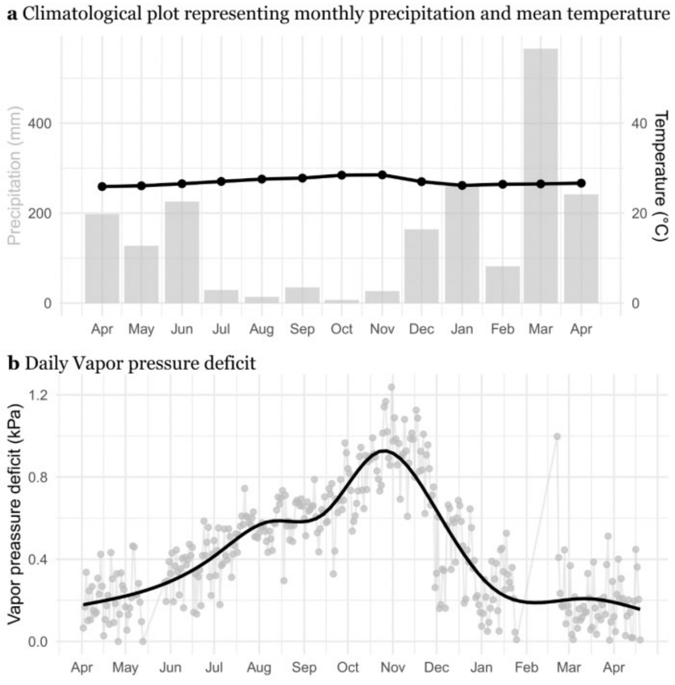

Figure S7. Meteorological data

Meteorological data from the study site from May 2023 to April 2024 (period during which tree hydraulics were monitored).

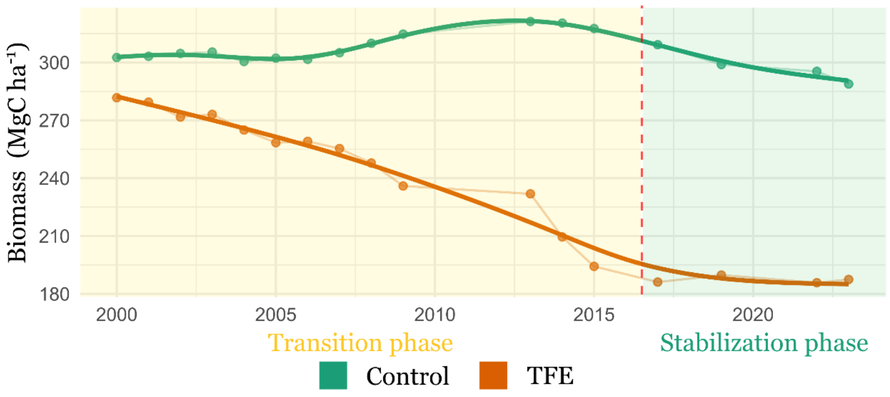

Figure S8. Biomass calculated from diameter tapes.

Estimated above ground wood biomass for Control and Throughfall Exclusion (TFE) plots during the whole drought experiment period (from 2002 to 2023) calculated from diameter measured by measuring tapes. Tendency lines are reported using general additive models. Red dotted line represents the approximate time at which the TFE plot changed from transition to stabilisation phase (i.e., stabilisation of biomass). The two phases are also represented by background colour.

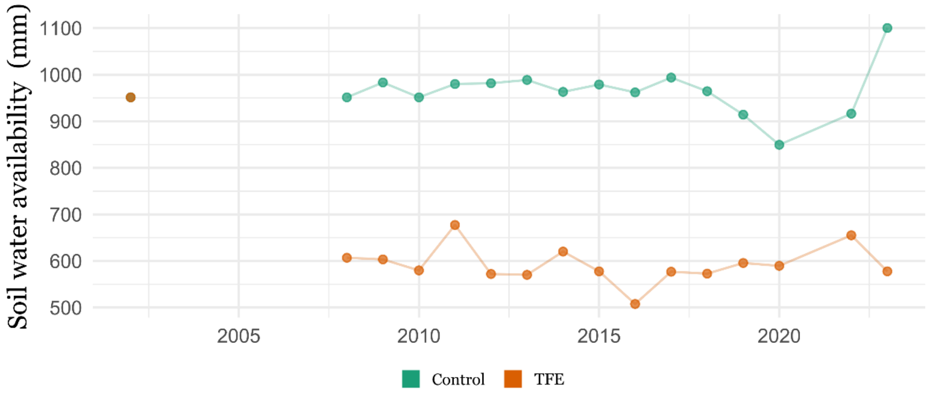

Figure S9. Soil water availability time series.

Soil water availability estimated as the 95% quantile of the annual water content (mm) in the top 4m of soil for Control and Throughfall Exclusion (TFE) plots during the whole drought experiment period (from 2002 to 2023).

Table S1. Sampled individuals.

ID	Size class	Plot	Species	DBH (cm)
**Control_312**	Intermediate	Control	*Pouteria cladantha*	28.65
**Control_354**	Intermediate	Control	*Manilkara bidentata*	51.41
**Control_359**	Intermediate	Control	*Micropholis venulosa*	34.70
**Control_211**	Intermediate	Control	*Licania octandra*	29.92
**Control_216**	Intermediate	Control	*Vouacapoua americana*	44.25
**Control_262**	Intermediate	Control	*Eschweilera coriacea*	40.11
**Control_279**	Intermediate	Control	*Rinorea guianensis*	17.19
**Control_218**	Intermediate	Control	*Swartzia racemosa*	29.44
**Control_316**	Large	Control	*Swartzia racemosa*	60.16
**Control_259**	Large	Control	*Manilkara paraensis*	77.35
**Control_215**	Large	Control	*Pouteria cladantha*	70.66
**Control_308**	Large	Control	*Pseudopiptadenia suaveolens*	87.22
**Control_220**	Large	Control	*Goupia glabra*	67.80
**Control_313**	Small	Control	*Licania octandra*	22.44
**Control_315**	Small	Control	*Pouteria decorticans*	17.83
**Control_317**	Small	Control	*Vouacapoua americana*	20.53
**Control_357**	Small	Control	*Eschweilera grandiflora*	15.92
**Control_264**	Small	Control	*Micropholis venulosa*	29.28
**Control_249**	Small	Control	*Protium tenuifolium*	12.41
**Control_256**	Small	Control	*Pouteria decorticans*	15.60
**Control_322**	Small	Control	*Vouacapoua americana*	26.42
**TFE_267**	Intermediate	TFE	*Quararibea guianensis*	63.18
**TFE_205**	Intermediate	TFE	*Eschweilera decolorans*	29.60
**TFE_207**	Intermediate	TFE	*Swartzia racemosa*	47.27
**TFE_111**	Intermediate	TFE	*Manilkara bidentata*	50.93
**TFE_178**	Intermediate	TFE	*Manilkara bidentata*	31.51
**TFE_200**	Intermediate	TFE	*Protium tenuifolium*	34.38
**TFE_82**	Intermediate	TFE	*Erisma uncinatum*	33.42
**TFE_168**	Intermediate	TFE	*Geissospermum sericeum*	37.24
**TFE_217**	Intermediate	TFE	*Eschweilera coriacea*	38.52
**TFE_266**	Large	TFE	*Trattinnickia burserifolia*	159.47
**TFE_270**	Large	TFE	*Erisma uncinatum*	70.98
**TFE_119**	Large	TFE	*Vouacapoua americana*	63.98
**TFE_169**	Large	TFE	*Manilkara bidentata*	58.57
**TFE_211**	Small	TFE	*Licania kunthiana*	29.28
**TFE_116**	Small	TFE	*Pouteria ramiflora*	24.83
**TFE_121.1**	Small	TFE	*Erisma uncinatum*	14.96
**TFE_122.1**	Small	TFE	*Protium pilosissimum*	11.46
**TFE_78**	Small	TFE	*Hymenolobium flavum*	
**TFE_213**	Small	TFE	*Manilkara huberi*	22.92
**TFE_214.1**	Small	TFE	*Pouteria decorticans*	10.50
**TFE_214.3**	Small	TFE	*Micropholis venulosa*	13.37

Table S2. Variance explained by genus and diameter.

Variance explained by diameter at the breast height (DBH) and genus for leaf water potential at midday and predawn (WP md, WP pd), maximum daily sap flow (Max. sap flow) and maximum daily stem water content (Max. stem wc) for the whole year and peak of the wet (May 2023) and dry (October 2023) season.

Response	Predictor	Variance explained
**WP md (whole year)**	DBH	0.01
**WP md (whole year)**	genus	0.1
**WP md (wet season)**	DBH	0.03
**WP md (wet season)**	genus	0.24
**WP md (dry season)**	DBH	0
**WP md (dry season)**	genus	0.27
**WP pd (whole year)**	DBH	0
**WP pd (whole year)**	genus	0.1
**WP pd (wet season)**	DBH	0.01
**WP pd (wet season)**	genus	0.02
**WP pd (dry season)**	DBH	0
**WP pd (dry season)**	genus	0.42
**Max. sap flow (all year)**	DBH	0
**Max. sap flow (all year)**	genus	0.28
**Max. sap flow (wet season)**	DBH	0
**Max. sap flow (wet season)**	genus	0.24
**Max. sap flow (dry season)**	DBH	0
**Max. sap flow (dry season)**	genus	0.57
**Max. stem wc (all year)**	DBH	0.01
**Max. stem wc (all year)**	genus	0.35
**Max. stem wc (wet season)**	DBH	0
**Max. stem wc (wet season)**	genus	0.31
**Max. stem wc (dry season)**	DBH	0
**Max. stem wc (wet season)**	genus	0.35
